# Formation and Regulation of Mitochondrial Membranes

**DOI:** 10.1155/2014/709828

**Published:** 2014-01-22

**Authors:** Laila Cigana Schenkel, Marica Bakovic

**Affiliations:** Department of Human Health and Nutritional Sciences, University of Guelph, Guelph, ON, Canada N1G 2W1

## Abstract

Mitochondrial membrane phospholipids are essential for the mitochondrial architecture, the activity of respiratory proteins, and the transport of proteins into the mitochondria. The accumulation of phospholipids within mitochondria depends on a coordinate synthesis, degradation, and trafficking of phospholipids between the endoplasmic reticulum (ER) and mitochondria as well as intramitochondrial lipid trafficking. Several studies highlight the contribution of dietary fatty acids to the remodeling of phospholipids and mitochondrial membrane homeostasis. Understanding the role of phospholipids in the mitochondrial membrane and their metabolism will shed light on the molecular mechanisms involved in the regulation of mitochondrial function and in the mitochondrial-related diseases.

## 1. Introduction

Mitochondria are involved in a wide range of cellular processes of importance for cell survival. The inner mitochondrial membrane is the active site for the electron transport chain and ATP production. Its integrity is crucial for mitochondrial function and depends on the supply of proteins and phospholipids. As one of the major classes of lipids in the lipid bilayer of cell and organelle membranes, phospholipids are responsible for maintaining both the structural integrity of a cell and spatial separation of subcellular compartments. The major classes of phospholipids found in the mitochondrial membrane are similar to other membranes such as phosphatidylcholine (PC) and phosphatidylethanolamine (PE), and some are exclusively components of mitochondrial membrane such as cardiolipin (CL) [1].

The interaction between phospholipids and proteins is important particularly in the inner mitochondrial membrane. A significant proportion of inner membrane-associated proteins are comprised of proteins involved in the oxidative phosphorylation and their activity depends on the phospholipid composition of the membrane. Changes in the phospholipid composition can affect mitochondrial respiration [2], which has been linked to a variety of human diseases such as Barth syndrome, ischemia, and heart failure [3, 4]. The phospholipid diversity in the mitochondrial membrane is also influenced by variation in length and degree of unsaturation of fatty acyl chain present within each class of phospholipid [5]. However the role of acyl chain composition of phospholipids in mitochondrial function is still poorly understood.

The maintenance of the phospholipid composition in the mitochondrial membranes is essential for mitochondrial function, structure, and biogenesis and relies on the metabolism of phospholipids, transport into mitochondria, and supply of lipids from the diet. In this review, we focus on the phospholipid biosynthesis, trafficking and degradation, and their regulation/remodeling by dietary lipids as well as their role in inner mitochondrial membrane integrity and function.

## 2. Lipid Composition of Mitochondrial Membranes

**Figure 1:**
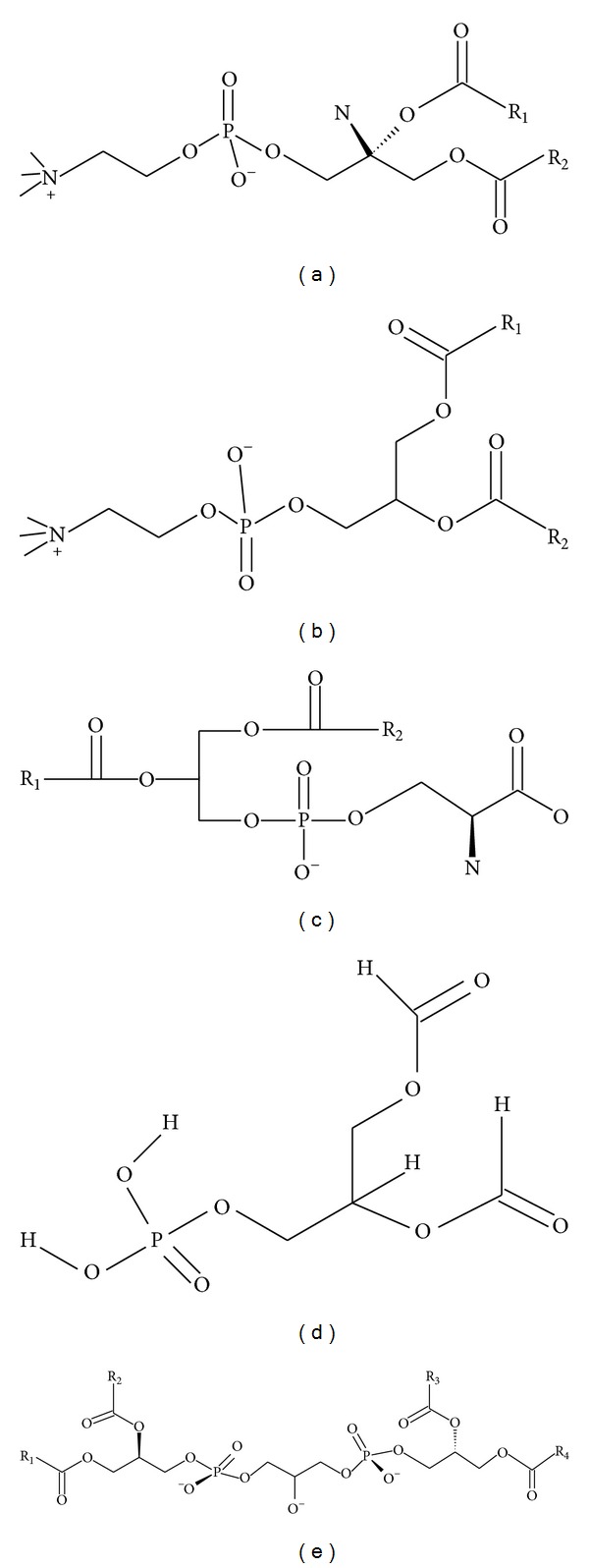
Structural formulas of key mitochondrial phospholipids. (a) Phosphatidylcholine—PC; (b) phosphatidylethanolamine—PE; (c) phosphatidic acid—PA; (d) phosphatidylserine—PS; (e) cardiolipin—CL.

Mitochondria have a structure distinct from that of other organelles since they contain two membranes: the outer mitochondrial membrane (OMM) and the inner mitochondrial membrane (IMM), which separates the intermembrane space (IMS) from the matrix. The composition of the mitochondrial membranes is similar to that of other membranes, however. The major phospholipids in the mitochondrial membranes are phosphatidylcholine (PC), phosphatidylethanolamine (PE), phosphatidylinositol (PI), phosphatidylserine (PS), and phosphatidic acid (PA), as to plasma membrane; and phosphatidylglycerol (PG) and cardiolipin (CL), are exclusively components of mitochondrial membrane (Figure 1). PC and PE are the most abundant phospholipids, comprising 40 and 30% of total mitochondrial phospholipids, respectively. PA and PS comprise 5% of the total mitochondrial phospholipids [6, 7]. Unlike plasma membrane, mitochondrial membranes contain high levels of cardiolipin (~15% of total phospholipids) and low levels of sphingolipids and cholesterol [8, 9].

The phospholipid CL not only has a role in maintaining membrane potential and architecture of the inner mitochondrial membrane, but also provides essential structural and functional support to several proteins involved in mitochondrial respiration. The unique structure of CL (Figure 1), which contains three glycerol backbones and four fatty acyl chains, makes it highly important for optimal function of the mitochondria [10]. The incorporation of four linoleic acid side chains in the same CL molecule renders it the primary target of the attack by free radicals, causing its peroxidation. Mitochondrial CL peroxidation appears to be an early event preceding the intrinsic apoptotic cell death [11]. Because its role in apoptosis and mitochondrial function, CL levels and CL peroxidation have been implicated in several human diseases such as atherosclerosis [12], cancer [13], Barth syndrome [14], and neurodegenerative disorders including Alzheimer's [15] and Parkinson's disease [16].

Another peculiarity of the IMM is that the amount of membrane-associated proteins is high. Some studies estimate the protein-lipid ratio of the inner membrane to be as high as 3 : 1 [17]. Among the IMM proteins, a significant portion of proteins comprises the oxidative phosphorylation system, which contains five multiprotein complexes. The importance of this system relies on its role in providing the cellular ATP molecules necessary for cell survival. The activity and stability of the oxidative phosphorylation proteins are affected by their interaction with phospholipids in the IMM. For example, the lack of cardiolipin in IMM was found to destabilize the complexes III and IV of the oxidative phosphorylation system [18, 19], illustrating the importance of CL for mitochondrial respiration. In addition, CL is also involved in the import and assembly of proteins. The majority of mitochondrial proteins are nuclearly encoded and require protein translocases in the mitochondrial membranes in order to be imported [20]. Several studies have shown that CL is essential for assembly and function of the translocases, allowing the integrity of the mitochondrial import machinery [21–23].

In addition to having a role in regulating protein import and activity, CL, as well as PE, is important for tubular mitochondrial morphology and membrane fusion [24–28]. Mitochondrial morphology depends on a balance between fusion and fission events. Mitochondrial fusion requires a fusogenic lipid PA which is generated by hydrolysis of CL by phospholipase D [26]. PE is another phospholipid required for the mitochondrial fusion. Disruption of the PE synthesis through PS decarboxylation pathway causes mitochondria fusion defects [25]. CL and PE accumulation within mitochondria are regulated by Ups1 and Ups2 proteins, respectively, which are involved in the phospholipid intramitochondrial trafficking (see Section 6). Ups1 and Ups2 affect the processing of OPA1 (or Mgm1 in yeast), an inner membrane dynamin-like GTPase that regulates membrane fusion [8]. It was suggested that impaired processing of Mgm1 could explain the defects in mitochondrial morphology with an altered membrane phospholipid composition [8]. A decrease in mitochondrial content of PE was found to alter mitochondrial morphology in yeast [27] and mammalian cells [25]. Joshi and colleagues have shown that CL and PE have overlapping functions in the mitochondrial fusion and that disruption of both PE and CL causes loss of mitochondrial membrane potential, and fragmentation of yeast mitochondria [27]. Fragmented mitochondria are associated with cardiomyopathy and Barth syndrome, showing the relevance of CL and PE role in the mitochondrial fusion. In this line, a yeast *crd1* mutant that does not synthetize CL exhibits multiple mitochondrial and cellular defects, including loss of mitochondrial DNA, decreased respiratory function and membrane potential and reduced cell viability at elevated temperatures as a consequence of the lack of CL [29]. Similar phenotypes were found in *psd1* yeast mutant, which has reduced mitochondrial PE [30]. Taken together, mitochondrial phospholipids CL and PE both regulate mitochondrial fusion/fission and biogenesis, which in turn are linked to the cellular homeostasis.

The phospholipid PA has important functions as a lipid anchor to recruit proteins involved in trafficking [31], lipid signaling [32], and fusion [33] to membrane surfaces. The regulation of mitochondria fusion and fission by PA was found to enable mitochondria to alter their morphology, to increase the efficiency of energy production, and to mark unhealthy mitochondria for autophagy [34]. In addition, PA can serve as substrate for production of the signaling lipids diacylglycerol (DAG) and lysophosphatidic acid. Mutations in the Lipin-1 gene, the PA phosphatase that generates DAG from PA, have been associated with metabolic and neurological diseases [35].

These evidences demonstrate the importance of the mitochondrial membrane phospholipids for the IMM architecture, the apoptosis, the activity of respiratory proteins, and the transport of proteins into the mitochondria. Because of their broad role in the mitochondrial function, phospholipid alterations have been associated with several diseases [36]. For example, cancer cells, which are characterized by alterations in bioenergetics and apoptosis, have altered mitochondrial phospholipid composition [37]. Cardiac pathologies result in a reduction of tetralinoleyl-CL, with consequent increase in hydrogen peroxide by the respiratory chain and apoptosis [38, 39]. Metabolic diseases, such as diabetes and nonalcoholic fatty live disease, have also been associated with decrease in mitochondrial CL and alteration of CL acyl chain remodeling [40, 41]. Another important aspect related to phospholipid composition is their change in aging. Studies have reported that aging is inversely related to the content of unsaturated phospholipids [42]. Loss of membrane fluidity resulting from lipid peroxidation, as well as decrease in mitochondrial CL content, and altered activity of the respiratory chain are also features of aging [43]. Therefore, the regulation of phospholipid homeostasis in mitochondrial membranes through their synthesis/degradation and transport into and out of the mitochondrial membrane plays a crucial role in maintaining cellular viability and health.

## 3. The Biosynthesis of the Major Membrane Phospholipids Occurs in the ER

**Figure 2:**
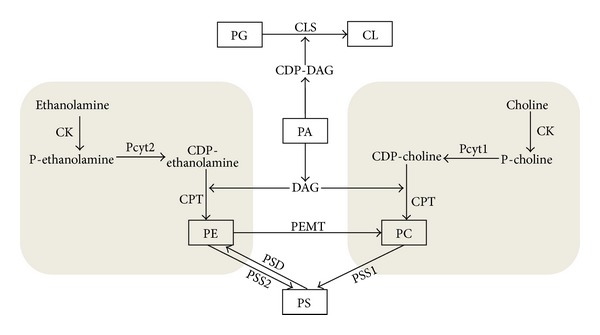
Pathways for the synthesis of phospholipids and their interconnection. The two branches of the Kennedy pathway are represented in grey. The key metabolites and enzymes catalysing the respective reactions are indicated. Abbreviations are as indicated in the main text.

Two major membrane bilayer phospholipids PC and PE are produced from choline and ethanolamine and the common lipid intermediate diacylglycerol in the process known as the *de novo* CDP-choline and CDP-ethanolamine Kennedy pathway [44, 45] (Figure 2). Both brunches of the pathway depend on the availability of the extracellular substrates, choline and ethanolamine, and on their entrance into the cells. Those substrates are polar molecules and have to be actively transported into the cells. The transport system for ethanolamine has not been extensively studied and remains poorly understood. Choline transport for PC synthesis is known to be mediated by a group of ubiquitous solute carriers of the SLC44A family, mainly by the member A1, also known as the choline transporter-like protein 1 (CTL1) and as the cell surface antigen CDw92 [46]. Immediately after entering the cells, choline and ethanolamine are phosphorylated by choline and ethanolamine kinases, of which CK*α* and -*β* and EK*α* and -*β* were identified and fully characterized in mammalian systems [47]. The kinase products, phosphocholine and phosphoethanolamine, are then coupled with CTP by the specific and the pathway regulatory enzymes CDP-phosphocholine and CDP phosphoethanolamine cytidylyltransferases (CCT/Pcyt1 and ECT/Pcyt2) to yield CDP-choline and CDP-ethanolamine, respectively, and to release inorganic pyrophosphate. In the final steps, the CDP-choline and the CDP-ethanolamine derivatives are condensed with diacylglycerol, catalysed by multiple DAG-choline and DAG-ethanolamine phosphotransferases (CPT, EPT, CEPT), to release CDP and to produce the bilayer forming phospholipids PC and PE at the endoplasmic reticulum (ER).

The major regulatory points for the PC and PE synthesis through Kennedy pathway are the slowest (rate-limiting) reactions governed by Pcyt1 and Pcyt2, respectively. Also, as in other metabolic pathways, the Kennedy pathway is regulated by the substrate (choline/ethanolamine and DAG) availabilities. The regulation of Pcyt1 and Pcyt2 is extensively studied and has historically being considered similar, but evidence is emerging that they are completely distinct and differently regulated [48–53].

The rate of PC synthesis is predominantly regulated by Pcyt1 activity [54]. Pcyt1 is activated by association with membranes and downregulated by phosphorylation [55]. The translocation of Pcyt1 into cell membranes is stimulated by fatty acids as well as anionic phospholipids [56–59]. They induce the Pcyt1 binding to membranes, because of their negatively charged head groups [59]. Thus, changes in the lipid composition of the membranes can modify Pcyt1 activity, which typically leads to stimulation of PC synthesis.

Yet another regulatory step for PC synthesis is the choline availability which is regulated by the protein-mediated transport at the level of the plasma membrane. The universal transporter CTL1 transports choline in various tissues [60–62] and is considered predominant choline transporter in nonneuronal cells [46, 60, 62, 63]. Recently, it has been found that the CTL1 expression, choline uptake, and PC synthesis could be directly regulated by choline deficiency [64] and saturated and unsaturated fatty acids (FAs) in skeletal muscle cells [65]. The availability of choline and the type of FAs regulate PC synthesis together with differently modifying choline transport, PC synthesis, and DAG/TAG homeostasis. In choline deficient cells, PC synthesis from choline was blunted, which led to an increased availability of DAG and FA for TAG synthesis [64]. Similar to choline deficiency, chronic exposure of muscle cells to palmitic acid reduced CTL1, choline uptake, and PC synthesis and increased DAG and TAG [65]. In contrast, oleic acid showed no effect on CTL1 and choline uptake; however, it increased PC synthesis at the level of Pcyt1 and elevated TAG content [65]. Oleic acid is a well-known stimulator of Pcyt1 membrane binding and activity [56–59].

The *de novo* synthesis of PE could be limited by the availability of substrates ethanolamine [66, 67] and DAG [68] or the enzymes EK [69] and Pcyt2 [53]. Based on the numerous radiolabeling studies and animal models, Pcyt2 is the main regulatory enzyme in the CDP-ethanolamine pathway. Heterozygous mice for the Pcyt2 gene (Pcyt2^+/−^) have reduced formation of CDP-ethanolamine, which limits the PE synthesis and increases the availability of DAG for TAG synthesis [70]. On the other hand, the overexpression of Pcyt2 could not accelerate PE synthesis when DAG availability for the last step in the pathway was limiting the PE formation [68], whereas EK overexpression accelerated PE synthesis in Cos-7 cells [69]. At low levels of ethanolamine, Pcyt2 limits the reaction rate, whereas at high levels of ethanolamine, EK limits the rate [71]. In ethanolamine deficient media, cells adapt the mitochondrial PS decarboxylation pathway to produce additional PE from serine (see Section 4) [72]. However, this is not the case in normal metabolism. Animal studies indicate that CDP-ethanolamine pathway is the major contributing pathway for the synthesis of PE [53].

In the liver, an alternative pathway utilizes PE to produce more PC and choline in a three-step methylation of PE by S-adenosylmethionine (SAM) catalysed by phosphatidylethanolamine-N-methyltransferase (PEMT). The PEMT pathway accounts for ~30% of the hepatic PC synthesis and is coregulated with the CDP-choline Kennedy pathway [73]. It is not known how the CDP-ethanolamine Kennedy pathway, which only makes PE *de novo*, is linked with the PE used in the PEMT pathway for the formation of PC. However, in the PEMT knockout mice, the CDP-choline pathway is upregulated, whereas during the PEMT overexpression in hepatoma cells, it is downregulated, showing a strong link of the PEMT pathway with the liver PC metabolism [74, 75]. As a backup pathway, PEMT supplies PC for the very-low-density lipoprotein (VLDL) assembly and for the bile production and is a source of choline for the betaine synthesis in mitochondria [76]. The liver methylation of PE also provides PC for PS synthesis by the base-exchange reaction catalysed by PS synthase 1 (PSS1). The newly formed PS could be then transformed into mitochondrial PE by PS decarboxylase (PSD), forming a specific liver PE cycle [77] (Figure 2). The liver cycle PE-PC-PS may be involved in the maintenance of phospholipid levels when *de novo* CDP-choline and CDP-ethanolamine pathways are impaired.

In mammals, PS could be only made from the preexisting phospholipids (PC and PE) and L-serine, catalysed by PS synthases 1 and 2 (PSS1 and PSS2) in the mitochondria associated membranes (MAM), a subfraction of the ER. MAM have been proposed to be a distinct domain of the ER that comes into close contact with OMM and thereby mediates the import of newly synthesized PS into mitochondria to be decarboxylated into PE. The PS decarboxylation by PSD to form mitochondrial PE is a key function of PS in mitochondria (see Section 4).

The synthesis of PS is regulated by phosphorylation of PS synthase in yeast and mammalian cells [78, 79]. The major mechanism for regulating PS synthesis in mammalian cells is a feedback mechanism whereby the activity of PS synthases 1 and 2 is regulated by the end-product, PS [77, 80, 81]. In addition, PSS1 and PSS2 differentially modulate phospholipid metabolism. Overexpression of PSS1 in hepatoma cells decreases the rate of PE synthesis via the CDP-ethanolamine pathway [82], whereas overexpression of PSS2 does not [83].

## 4. The Biosynthesis of Mitochondrial Phospholipids

The maintenance of the mitochondrial bilayer and of a defined composition of mitochondrial phospholipids relies on the organelle capacity to synthesize CL, PE, PG, and PA *in situ* and on the external supply of PC and PS, which are exclusively synthesized in the ER and MAM and must be imported into the mitochondria [8].

PE is made in mitochondria by decarboxylation of PS. This reaction is catalysed by the inner mitochondrial enzyme phosphatidylserine decarboxylase (PSD). Even though PE produced by the CDP-ethanolamine pathway can be imported to IMM, the PS decarboxylation provides the majority of mitochondrial PE [84]. PSD knockout mice have mitochondrial dysfunction and die in the embryonic phase [25]. In addition, the mitochondrial PE deficiency resulting from siRNA silencing of PSD in CHO cells alters mitochondrial morphology and affects the ATP production, oxygen consumption, and the activity of the electron-transport components [85].

Cardiolipin, an important phospholipid in the mitochondrial membranes, is synthesized via condensation of phosphatidylglycerol (PG) and CDP-diacylglycerol catalysed by cardiolipin synthase (CLS) in the matrix side of the inner mitochondrial membrane (Figure 2). PG is produced by a two-step reaction: glycerol-3-phosphate and CDP-diacylglycerol condense to phosphatidylglycerol phosphate, which is then dephosphorylated to PG. Lack of CLS reduces the activity of the oxidative phosphorylation system and the import of proteins into mitochondria in yeast [86], but interestingly, the fundamental functions of mitochondria remain intact [87]. The regulation of CL synthesis was found to involve multiple mechanisms, such as factors that affect mitochondrial biogenesis, matrix PH, and respiration [88]. Recently, Tam41 (translocator and maintenance protein 41), a component of the mitochondrial translocator system, was found to regulate CL synthesis [23]. *Tam41* mutant cells show an almost complete absence of CL and PG, suggesting that regulation may occur at the level of CDP-diacylglycerol (CDP-DAG) synthase. This study demonstrates that Tam41 is primarily required for PG and CL biosynthesis and that defects in protein import in Tam41 deficient cells are a consequence of the loss of PG and CL [23].

Finally, the phospholipid phosphatidic acid (PA) represents a branch-point for the synthesis of all phospholipids [89] (Figure 2). PA may be converted to CDP-DAG by the CDP-DAG synthase for the synthesis of PG, PS, and CL in a reaction catalysed by CTP:PA cytidylyltransferase [90]. Alternatively, PA may be converted to DAG, which is a substrate for the synthesis of PE and PC, in a reaction catalysed by PA phosphatases such as Lipin-1 [35, 91]. The PA phosphatases are one of the most highly regulated enzymes in lipid metabolism. Their activity is governed by phosphorylation, association with membranes, and modulation by components of lipid metabolism, such as CL and CDP-DAG [92, 93].

The synthesis of PA occurs by two acylation steps; first glycerol-3-phosphate is converted to 1-acylglycerol-3-phosphate (lysophosphatidic acid) by glycerol-3-phosphate acyltransferase. The 1-acyl-sn-glycerol-3-phosphate product is then acylated to 1,2-diacyl-sn-glycerol-3-phosphate (phosphatidic acid) by 1-acyl-sn-glycerol-3-phosphate acyltransferase [94]. Studies have indicated that both lysophosphatidic acid and phosphatidic acid are synthesized on the outer surface of the mitochondrial outer membrane and then phosphatidic acid moves to the inner mitochondrial membrane where it serves as a precursor for cardiolipin biosynthesis [95]. PA can also be generated via phosphorylation of DAG through the action of a large family of DAG kinases (DAGK) or by hydrolysis of the phospholipids PC and CL by phospholipase D (see Section 8). In turn, PA can be metabolized to lysophosphatidic acid by phospholipase A2 or dephosphorylated to DAG by Lipin-1.

## 5. Trafficking of Phospholipids into and out of Mitochondria

Intracellular trafficking of phospholipids plays a crucial role in phospholipid homeostasis and provides phospholipids for cell and organelle membranes, including the OMM and IMM. Since most phospholipids, such as PE, PS, and PC, are synthesized in the ER, they have to be imported into the mitochondria. The import of PE produced by the CDP-ethanolamine pathway into IMM was found to be effective in CHO cells, HeLa cells, and yeast [96–98]. However, most of the PE in the mitochondria derives from the PS decarboxylation [85]. The synthesis of PE from PS in the IMM requires the translocation of PS from its synthesis sites in the ER. In addition, several studies have shown that PE formed from PS in the mitochondria could be exported out of this organelle [85, 97]. The transport of PS-derived PE out of the mitochondria was found to be affected by the rate of PE synthesis via the CDP-ethanolamine pathway and to be driven by a concentration gradient [97].

The mechanism for transport of phospholipids into and out of the mitochondrial membranes has been proposed to involve transient membrane contact sites. These contact sites occur in various organelles including the ER and mitochondria and may be involved in the trafficking of PS into and PE out of the mitochondria [99, 100]. Recent studies found that the ER membrane is physically tethered to the OMM by the ER-mitochondria encounter structure (ERMES) [101]. The ERMES is composed of a five-protein complex resident of both ER and mitochondria [101]. The main function of ERMES is to act as a mechanical link between the ER and mitochondria and to provide phospholipid exchange between these organelles [102, 103]. A defective ERMES complex alters phospholipid levels in the mitochondrial membranes and causes mitochondrial morphological defects [103, 104]. However, the flow of phospholipids between ER and mitochondria is not completely abolished in ERMES deficient cells, suggesting that additional ERMES-independent pathways for phospholipids transport must also exist [102].

Lipid transfer proteins, such as PC-transfer protein and nonspecific lipid transfer protein, have been shown to transfer phospholipids into the plasma membrane and may be also involved in the exchange of phospholipids between organelle membranes [105]. However, no transfer protein mediating PC, PS, and PE trafficking into/out of mitochondria has been established to date.

The rate of PS import to and PE export from the mitochondria is regulated by the acyl chain composition and by the metabolic rate [97]. Labeling studies showed that polyunsaturated PS species are preferentially decarboxylated, that is, imported to IMM [97]. In turn, PE derived from decarboxylation of PS contains the major polyunsaturated species as well as monounsaturated 36 : 1, whereas PE produced via the CDP-ethanolamine pathway contains several mono- and di-unsaturated species. The PE species composition of IMM and ER shows that most of PE in IMM derives from imported PS, while most of PE in ER is synthesized via the CDP-ethanolamine pathway [97]. In addition, Kainu and colleagues found that increasing the PE synthesis by CDP-ethanolamine pathway reduces the export of PS-derived PE from IMM. Similarly, the translocation of PS to mitochondria is coupled to its synthesis in the ER. However, the rate of transport of newly synthesized PS to mitochondria does not directly correlate with their rate of synthesis but depends on its hydrophobicity [97].

## 6. Intramitochondrial Trafficking of Phospholipids

The transbilayer movements between the mitochondrial leaflets must exist to allow the trafficking and accumulation of phospholipids, either when imported from ER or when synthesized at the IMM. Several studies have shown that the import of PS into mitochondria is mediated by the membrane contact between MAM, a subfraction of the ER, and OMM [84, 85, 97, 106, 107]. The PS must then translocate across the OMM and subsequently be delivered from the inner leaflet of the OMM, across the intermembrane space, to the outer leaflet of the IMM, which is the active site for PS decarboxylation to PE [108]. The mechanisms underlying the intramitochondrial lipid movement have been also suggested to occur via membrane contact sites [99, 109]. The model that best describes the intramitochondrial transport of PS involves pores in the OMM that lead to its movement from the outer leaflet of the OMM to the inner leaflet of the OMM. PS then diffuses further to the IMM along lipid bridges, which are analogous to the membrane contact sites, joining the two membranes [110]. Once in the IMM, PS can be converted to PE. The PE produced in the IMM can then diffuse back to the outer monolayer of OMM along a reverse route.

Phospholipid scramblases (PLS), members of the family of transmembrane lipid transporters known as flippases, are enzymes responsible for bidirectional movement of phospholipids between two compartments. Of this family, PLS3 was identified in mitochondria and it modulates translocation of CL from the IMM to the OMM, affecting mitochondrial structure, respiration, and apoptosis [111]. It is not known if PLS3 regulates transport of other phospholipids. The intermembrane space proteins Ups1 and Ups2 are responsible for CL and PE trafficking between the outer and inner mitochondrial membranes [112]. Loss of intramitochondrial CL trafficking in Ups1 deficient cells alters mitochondrial phospholipid composition and morphology, similar to ERMES deficient cells [112]. Mdm35, a common binding partner of Ups1 and Ups2 in the intermembrane space, also provides a coordinated regulation of PE and CL trafficking by these conserved regulatory proteins [113].

Therefore, the trafficking of phospholipids into mitochondria and intramitochondrial space provides this organelle with newly synthetized phospholipids, which are either essential for mitochondrial membrane composition or precursor for the synthesis of specific mitochondrial phospholipids. The balance in phospholipid trafficking, together with their synthesis and degradation, is essential for the maintenance of phospholipid homeostasis. However, not only the class of phospholipids accumulated in the mitochondrial membrane, but also the fatty acyl chain present within phospholipids is important for mitochondrial structure and function, which will be described in the next section.

## 7. Remodeling of Mitochondrial Phospholipids and the Role of Dietary Lipids

After the *de novo* synthesis of phospholipids, many of them undergo acyl chain remodeling, known as Lands' cycle. The variation in chain length and degree of unsaturation of fatty acids present within phospholipids contributes to the diversity of mitochondrial membrane phospholipids, which is important for the biophysical properties of the membrane [114, 115]. In addition, activation of enzymes in the inner mitochondrial membrane requires acyl chain remodeling. For example, impairment of CL remodeling may interfere with assembly and stability of the respiratory chain proteins [115]. Conventionally, acyl chain remodeling involves the family of phospholipases A (PLAs), which catalyse the removal of an acyl chain from the glycerol moiety, and transacylases, which mediate reacylation with different FAs [114, 116].

Remodeling of cardiolipin has been extensively studied due to its role in the functionality of the mitochondria. CL remodeling involves tafazzin, a transacylase which generates specific patterns of CL species [117]. Deficiency of tafazzin alters CL composition and is related to dramatic changes in mitochondrial morphology and to Barth syndrome [118]. Barth syndrome is an X-linked recessive disease characterized by decreased levels of CL due to mutation in the tafazzin gene [119]. In Barth patients, a single molecular species, namely, tetralinoleoyl-cardiolipin, is missing, whereas other cardiolipins are either unaffected or even increased. This is caused by a reduced incorporation of linoleic acid (C18:2) into CL because of impaired FA remodeling by tafazzin [118, 120]. Changes in the cardiolipin FA composition in Barth syndrome may also interfere with assembly and stability of the oxidative phosphorylation complexes.

Another enzyme involved in cardiolipin remodeling is acyl coenzyme A thioesterase (Them5). Mice lacking Them5 have an altered mitochondrial morphology and function in hepatocytes [121]. Similarly, deficiency of lipocalin-2, a lipid transfer protein, alters the fatty acid composition of the PE, PC, and PS as well as the amount of mitochondrial cardiolipin in the mouse heart [122]. Lipocalin was found to reduce the linoleic acid (C18:2) content in phospholipids and to adversely affect mitochondrial function and energy production [122]. Indeed, cardiolipin enriched with symmetric linoleic acid allows for optimal function of the mitochondria [123].

The fatty acid composition of the diet can additionally modify the acyl chain remodeling of phospholipids and the mitochondrial function. In one study, mice exposure to rapeseed oil-rich diet showed altered mitochondrial membrane phospholipid composition in terms of both the type of acyl chains within the phospholipids and the proportions of phospholipid classes. As a result of altered mitochondrial membrane composition, these mice showed altered hepatic mitochondrial bioenergetics [124]. Guderley and collaborators showed that in trout mitochondria the changes in membrane phospholipid characteristics, including the acyl-chain composition, follow the patterns of fatty acids present in the diet. In this same study, the respiratory capacity of the red muscle mitochondria was altered by the different diets and was found to be higher in the diet rich in polyunsaturated lipids [125]. Several other studies in mammals have also demonstrated that dietary changes modify the FA composition of the major mitochondrial phospholipids [126–128] and, in particular, the molecular species associated with mitochondrial CL [57]. Following a diet deficient in linoleic acid (C18:2), an essential fatty acid, the rat heart showed a significant decrease in tetralinoleoyl CL, which affected mitochondrial oxygen consumption [127, 129]. Conversely, dietary supplementation with linoleic acid restored tetralinoleoyl CL in cultured fibroblasts from Barth syndrome patients and elevated CL levels [130].

The composition of mitochondrial CL was found to be altered by both quantity and quality of the dietary fat [131]. In hepatocytes, the composition of mitochondrial CL was altered in rats fed 30% fat diets in comparison to rats fed 5% fat diets. Also, the content of monounsaturated fatty acid (MUFA) and n-3 polyunsaturated fatty acids (PUFA) was increased with fish oil-rich diet in comparison to basal diet, both at 5% and 30% of fat in diet. Both total CL content and its C18:2 content were increased with liver steatosis and correlated to the activity of the ATP synthase [131]. Recently, a mitochondrial lipidomics analysis showed that the FA composition of the CL pool is not directly related to the presence of a given FA in the diet, but there is a selection for individual FA chains to be incorporated into the CL pool [132]. For example, the incorporation of 18:2 was found to be similar, despite diets varying ~4-fold in this essential FA.

The n-3 PUFA content of the plasma membrane in different mouse tissues had the greatest sensitivity to changes in dietary fatty acids [133]. The fact that both n-6 and n-3 PUFA classes cannot be synthesised *de novo* by mammals suggests that the composition of membrane phospholipids may be strongly influenced by the ratio and abundance of n-6 and n-3 PUFAs in the diet. Several studies have shown that increasing the PUFA content of the diet increases the metabolic rate [134–136]. For example, treatment of rats with n-3 PUFA decreased proton leakage from the respiratory chain and this was related to the incorporation of PUFAs into mitochondrial PC, PE, and CL [137]. Treatment with docosahexaenoic acid (DHA), but not eicosapentaenoic acid (EPA), profoundly alters fatty acid composition of mitochondrial phospholipids in the rat heart, decreasing arachidonic acid and increasing DHA content. The increased DHA content delayed the mitochondrial permeability transition pore (MPTP) opening, which is associated with apoptosis and myocardial damage during ischemia [127]. DHA incorporation into CL was also found to regulate mitochondrial lipid-protein clustering, which alters several aspects of mitochondrial function [17]. Taken collectively, these results suggest that increasing the level of PUFA in the diet changes the composition of mitochondrial membrane phospholipids and consequently may alter mitochondrial capacity and function.

The response to dietary fatty acids also varies according to the phospholipid class. PC was found to be more responsive to variation in dietary MUFA content than the other phospholipid classes, whereas PE was more responsive than PC to both dietary n-6 PUFA and the n-3 PUFA [133]. Interestingly, Pcyt2 deficient mice, which have a decreased rate of PE synthesis, are PUFA deficient and have specifically modified FA composition in PE and in TAG [70]. Therefore, change in the phospholipid fatty acyl chain composition relies not only on the dietary fatty acid profile but also on the classes of phospholipids affected.

Taken together, these studies demonstrated that disturbance of enzymes involved in phospholipid remodeling, as well as changes in the dietary fatty acids, may alter the phospholipid composition of the mitochondrial membrane. Dietary interventions that are able to influence mitochondrial membrane phospholipids, hence modifying its physical properties, respiration, and other processes such as MPTP, are emerging as novel therapeutic strategies [124]. Therapies addressed to mitochondrial phospholipids have been suggested as potentially useful to treat pathologies, such as cancer, cardiovascular and neurodegenerative diseases, obesity, and metabolic disorders [124].

## 8. Degradation of Mitochondrial Phospholipids

Many phospholipids have a rapid turnover, indicating that their degradation plays an important role in membrane homeostasis. The phospholipid degradation is mainly catalysed by nonlysosomal phospholipases. These phospholipases are divided into three classes based on the bound they cleave: phospholipases A (PLAs), phospholipases C (PLCs), and phospholipases D (PLD).

PLAs release the fatty acid in the *sn-*1 and *sn-*2 position of the glycerol moiety generating lysophospholipid and free FA. The lysophospholipid is reacylated to generate new phospholipid, the mechanism known as FA remodeling, or it is degraded by a lysophospholipase. Several studies suggest that PLAs mediate much of the phospholipids turnover [138, 139]. The PLA2 family, which hydrolyses FA bound at the *sn*-2 position of phospholipid, has been related to turnover and remodeling of phospholipids as well as to membrane homeostasis [140, 141]. iPLA2*γ*, a component of the Ca^2+^-independent PLA2 family, is preferentially distributed in the mitochondria. Because of the association of iPLA2*γ* with mitochondrial membranes, this enzyme may be involved in integrating phospholipid and energy metabolism. Mice null for iPLA2*γ* display abnormal mitochondrial function with a dramatic decrease in oxygen consumption [142]. Thus, iPLA2*γ* is essential for maintaining bioenergetic mitochondrial function through regulation of mitochondrial membrane phospholipid homeostasis. Because, its role in cardiolipin remodeling, the hippocampus of iPLA2*γ* null mice shows an elevated content of mitochondrial cardiolipin with an altered chain length composition [142].

Phospholipases C (PLCs) hydrolyse the bond between glycerol backbone and phosphate to yield DAG and phosphorylated head group. PLCs are generally involved in generation of second messengers for signalling events [143], but they also play a role in the turnover of PC and PE [144, 145].

Phospholipases D hydrolyse the bond between phosphate and the head group to yield PA and free head group. Both classical phospholipase D (PLD) family members found on many cytoplasmic membrane surfaces and MitoPLD, the PLD family member found on the mitochondrial surface, can generate PA via hydrolysis of the phospholipids PC and CL [26]. MitoPLD-generated PA regulates mitochondrial shape through facilitating mitochondrial fusion [146]. Mitochondrial fusion and fission are very important in maintaining mitochondrial and cellular function, and morphological changes of the mitochondria are linked to cell apoptosis and neurodegenerative disease [146]. For example, in brains of Alzheimer's disease patients, PLD1 is upregulated in the mitochondrial membrane, which affects the composition of mitochondrial membrane phospholipids [147].

The involvement of phospholipases in phospholipid homeostasis is also demonstrated by PLD-like enzymes that catalyse PS synthesis by base-exchange reactions. PS synthases 1 and 2 (PSS1/2), the PLD-like enzymes, mainly catalyse the exchange of the head group of PE or PC for serine rather than the exchange of the serine head group of PS for choline or ethanolamine. Thus, PLD and PLD-like enzymes could play an important role in different phospholipid homeostasis [84].

## 9. Conclusion

The identification of a link between phospholipids and proteins in the mitochondrial membrane has enhanced our understanding of mitochondrial morphology and function. The regulation of synthesis, trafficking, and degradation of phospholipids is essential to maintain phospholipid homeostasis in the mitochondria. Remodeling of phospholipid in the mitochondria can be modified by dietary fatty acids, which contribute to mitochondrial membrane integrity. However, the molecular mechanism for the coordination of synthesis, degradation, trafficking, and remodeling of phospholipids is still an active area of research. For example, how is the synthesis and degradation interconnected? Which are the signals for phospholipid transport into and out of mitochondria? How can diet be used to modulate phospholipid homeostasis in health and disease states? Undoubtedly, many discoveries will be achieved in a few years.

## References

[1] Zinser E, Sperka-Gottlieb CDM, Fasch E-V, Kohlwein SD, Paltauf F, Daum G (1991). Phospholipid synthesis and lipid composition of subcellular membranes in the unicellular eukaryote *Saccharomyces cerevisiae*. *Journal of Bacteriology*.

[2] Ohtsuka T, Nishijima M, Akamatsu Y (1993). A somatic cell mutant defective in phosphatidylglycerophosphate synthase, with impaired phosphatidylglycerol and cardiolipin biosynthesis. *Journal of Biological Chemistry*.

[3] Joshi AS, Zhou J, Gohil VM, Chen S, Greenberg ML (2009). Cellular functions of cardiolipin in yeast. *Biochimica et Biophysica Acta*.

[4] Chicco AJ, Sparagna GC (2007). Role of cardiolipin alterations in mitochondrial dysfunction and disease. *American Journal of Physiology: Cell Physiology*.

[5] Houtkooper RH, Turkenburg M, Poll-The BT (2009). The enigmatic role of tafazzin in cardiolipin metabolism. *Biochimica et Biophysica Acta*.

[6] Colbeau A, Nachbaur J, Vignais PM (1971). Enzymac characterization and lipid composition of rat liver subcellular membranes. *Biochimica et Biophysica Acta*.

[7] Zinser E, Daum G (1995). Isolation and biochemical characterization of organelles from the yeast, *Saccharomyces cerevisiae*. *Yeast*.

[8] Osman C, Voelker DR, Langer T (2011). Making heads or tails of phospholipids in mitochondria. *Journal of Cell Biology*.

[9] Houtkooper RH, Vaz FM (2008). Cardiolipin, the heart of mitochondrial metabolism. *Cellular and Molecular Life Sciences*.

[10] Yin H, Zhu M (2012). Free radical oxidation of cardiolipin: chemical mechanisms, detection and implication in apoptosis, mitochondrial dysfunction and human diseases. *Free Radical Research*.

[11] Kagan VE, Tyurin VA, Jiang J (2005). Cytochrome c acts as a cardiolipin oxygenase required for release of proapoptotic factors. *Nature Chemical Biology*.

[12] Berliner JA, Leitinger N, Tsimikas S (2009). The role of oxidized phospholipids in atherosclerosis. *Journal of Lipid Research*.

[13] Hammad LA, Wu G, Saleh MM (2009). Elevated levels of hydroxylated phosphocholine lipids in the blood serum of breast cancer patients. *Rapid Communications in Mass Spectrometry*.

[14] Barth PG, Scholte HR, Berden JA (1983). An X-linked mitochrondrial disease affecting cardiac muscle, skeletal muscle and neutrophil leucocytes. *Journal of the Neurological Sciences*.

[15] Montine TJ, Montine KS, McMahan W, Markesbery WR, Quinn JF, Morrow JD (2005). F2-isoprostanes in Alzheimer and other neurodegenerative diseases. *Antioxidants and Redox Signaling*.

[16] Porter FD, Scherrer DE, Lanier MH (2010). Cholesterol oxidation products are sensitive and specific blood-based biomarkers for Niemann-Pick C1 disease. *Science Translational Medicine*.

[17] Shaikh SR, Brown DA (2012). Models of plasma membrane organization can be applied to mitochondrial membranes to target human health and disease with polyunsaturated fatty acids. *Prostaglandins Leukotrienes and Essential Fatty Acids*.

[18] Pfeiffer K, Gohil V, Stuart RA (2003). Cardiolipin stabilizes respiratory chain supercomplexes. *Journal of Biological Chemistry*.

[19] Claypool SM, Oktay Y, Boontheung P, Loo JA, Koehler CM (2008). Cardiolipin defines the interactome of the major ADP/ATP carrier protein of the mitochondrial inner membrane. *Journal of Cell Biology*.

[20] Schmidt O, Pfanner N, Meisinger C (2010). Mitochondrial protein import: from proteomics to functional mechanisms. *Nature Reviews Molecular Cell Biology*.

[21] Gallas MR, Dienhart MK, Stuart RA, Long RM (2006). Characterization of Mmp37p, a *Saccharomyces cerevisiae* mitochondrial matrix protein with a role in mitochondrial protein import. *Molecular Biology of the Cell*.

[22] Tamura Y, Harada Y, Yamano K (2006). Identification of Tam41 maintaining integrity of the TIM23 protein translocator complex in mitochondria. *Journal of Cell Biology*.

[23] Kutik S, Rissler M, Guan XL (2008). The translocator maintenance protein Tam41 is required for mitochondrial cardiolipin biosynthesis. *Journal of Cell Biology*.

[24] Kawasaki K, Kuge O, Chang S-C (1999). Isolation of a Chinese hamster ovary (CHO) cDNA encoding phosphatidylglycerophosphate (PGP) synthase, expression of which corrects the mitochondrial abnormalities of a PGP synthase-defective mutant of CHO-K1 cells. *Journal of Biological Chemistry*.

[25] Steenbergen R, Nanowski TS, Beigneux A, Kulinski A, Young SG, Vance JE (2005). Disruption of the phosphatidylserine decarboxylase gene in mice causes embryonic lethality and mitochondrial defects. *Journal of Biological Chemistry*.

[26] Choi S-Y, Huang P, Jenkins GM, Chan DC, Schiller J, Frohman MA (2006). A common lipid links Mfn-mediated mitochondrial fusion and SNARE-regulated exocytosis. *Nature Cell Biology*.

[27] Joshi AS, Thompson MN, Fei N, Huttemann M, Greenberg ML (2012). Cardiolipin and mitochondrial phosphatidylethanolamine have overlapping functions in mitochondrial fusion in *Saccharomyces cerevisiae*. *Journal of Biological Chemistry*.

[28] Claypool SM, Boontheung P, McCaffery JM, Loo JA, Koehler CM (2008). The cardiolipin transacylase, tafazzin, associates with two distinct respiratory components providing insight into Barth syndrome. *Molecular Biology of the Cell*.

[29] Zhong Q, Gohil VM, Ma L, Greenberg ML (2004). Absence of cardiolipin results in temperature sensitivity, respiratory defects, and mitochondrial DNA instability independent of pet56. *Journal of Biological Chemistry*.

[30] Birner R, Nebauer R, Schneiter R, Daum G (2003). Synthetic lethal interaction of the mitochondrial phosphatidylethanolamine biosynthetic machinery with the prohibitin complex of *Saccharomyces cerevisiae*. *Molecular Biology of the Cell*.

[31] Yang J-S, Gad H, Lee SY (2008). A role for phosphatidic acid in COPI vesicle fission yields insights into Golgi maintenance. *Nature Cell Biology*.

[32] Huang H, Gao Q, Peng X (2011). PiRNA-associated germline nuage formation and spermatogenesis require MitoPLD profusogenic mitochondrial-surface lipid signaling. *Developmental Cell*.

[33] Nakanishi H, de Los Santos P, Neiman AM (2004). Positive and negative regulation of a SNARE protein by control of intracellular localization. *Molecular Biology of the Cell*.

[34] Chen H, Chomyn A, Chan DC (2005). Disruption of fusion results in mitochondrial heterogeneity and dysfunction. *Journal of Biological Chemistry*.

[35] Han G-S, Carman GM (2010). Characterization of the human LPIN1-encoded phosphatidate phosphatase isoforms. *Journal of Biological Chemistry*.

[36] Monteiro JP, Oliveira PJ, Jurado AS (2013). Mitochondrial membrane lipid remodeling in pathophysiology: a new target for diet and therapeutic interventions. *Progress in Lipid Research*.

[37] Kiebish MA, Han X, Cheng H, Chuang JH, Seyfried TN (2008). Cardiolipin and electron transport chain abnormalities in mouse brain tumor mitochondria: lipidomic evidence supporting the Warburg theory of cancer. *Journal of Lipid Research*.

[38] Chen Q, Lesnefsky EJ (2006). Depletion of cardiolipin and cytochrome c during ischemia increases hydrogen peroxide production from the electron transport chain. *Free Radical Biology and Medicine*.

[39] Reibel DK, O’Rourke B, Foster KA (1986). Altered phospholipid metabolism in pressure-overload hypertrophied hearts. *American Journal of Physiology: Heart and Circulatory Physiology*.

[40] Han X, Yang J, Cheng H, Yang K, Abendschein DR, Gross RW (2005). Shotgun lipidomics identifies cardiolipin depletion in diabetic myocardium linking altered substrate utilization with mitochondrial dysfunction. *Biochemistry*.

[41] Petrosillo G, Portincasa P, Grattagliano I (2007). Mitochondrial dysfunction in rat with nonalcoholic fatty liver. Involvement of complex I, reactive oxygen species and cardiolipin. *Biochimica et Biophysica Acta*.

[42] Pamplona R, Barja G, Portero-Otín M (2002). Membrane fatty acid unsaturation, protection against oxidative stress, and maximum life span: a homeoviscous-longevity adaptation?. *Annals of the New York Academy of Sciences*.

[43] Ames BN, Shigenaga MK, Hagen TM (1995). Mitochondrial decay in aging. *Biochimica et Biophysica Acta*.

[44] Kennedy EP (1957). Biosynthesis of phospholipides. *Federation Proceedings*.

[45] Kennedy EP, Weiss SB (1956). The function of cytidine coenzymes in the biosynthesis of phospholipides. *Journal of Biological Chemistry*.

[46] Michel V, Yuan Z, Ramsubir S, Bakovic M (2006). Choline transport for phospholipid synthesis. *Experimental Biology and Medicine*.

[47] Aoyama C, Yamazaki N, Terada H, Ishidate K (2000). Structure and characterization of the genes for murine choline/ethanolamine kinase isozymes *α* and *β*. *Journal of Lipid Research*.

[48] Vance JE, Vance DE (2004). Phospholipid biosynthesis in mammalian cells. *Biochemistry and Cell Biology*.

[49] Vermeulen PS, Tijburg LBM, Geelen MJH, van Golde LMG (1993). Immunological characterization, lipid dependence, and subcellular localization of CTP:phosphoethanolamine cytidylyltransferase purified from rat liver. Comparison with CTP:phosphocholine cytidylyltransferase. *Journal of Biological Chemistry*.

[50] Nakashima A, Hosaka K, Nikawa J-I (1997). Cloning of a human cDNA for CTP-phosphoethanolamine cytidylyltransferase by complementation in vivo of a yeast mutant. *Journal of Biological Chemistry*.

[51] Tijberg LBM, Geelen MJH, van Golde MG (1989). Biosynthesis of phosphatidylethanolamine via the CDP-ethanolamine route is an important pathway in isolated rat hepatocytes. *Biochemical and Biophysical Research Communications*.

[52] Tijburg LBM, Geelen MJH, van Golde LMG (1989). Regulation of the biosynthesis of triacylglycerol, phosphatidylcholine and phosphatidylethanolamine in the liver. *Biochimica et Biophysica Acta*.

[53] Bakovic M, Fullerton MD, Michel V (2007). Metabolic and molecular aspects of ethanolamine phospholipid biosynthesis: the role of CTP:phosphoethanolamine cytidylyltransferase (Pcyt2). *Biochemistry and Cell Biology*.

[54] Sugimoto H, Banchio C, Vance DE (2008). Transcriptional regulation of phosphatidylcholine biosynthesis. *Progress in Lipid Research*.

[55] Pelech SL, Vance DE (1982). Regulation of rat liver cytosolic CTP:phosphocholine cytidyltransferase by phosphorylation and dephosphorylation. *Journal of Biological Chemistry*.

[56] Weinhold PA, Charles LG, Feldman DA (1991). Microsomal CTP:choline phosphate cytidylyltransferase: kinetic mechanism of fatty acid stimulation. *Biochimica et Biophysica Acta*.

[57] Weinhold PA, Charles L, Rounsifer ME, Feldman DA (1991). Control of phosphatidylcholine synthesis in Hep G2 cells: effect of fatty acids on the activity and immunoreactive content of choline phosphate cytidylyltransferase. *Journal of Biological Chemistry*.

[58] Wang Y, MacDonald JIS, Kent C (1993). Regulation of CTP:phosphocholine cytidylyltransferase in HeLa cells. Effect of oleate on phosphorylation and intracellular localization. *Journal of Biological Chemistry*.

[59] Cornell R, Vance DE (1987). Translocation of CTP:phosphocholine cytidyltransferase from cytosol to membranes in HeLa cells: stimulation by fatty acid, fatty alcohol, mono- and diacylglycerol. *Biochimica et Biophysica Acta*.

[60] Yuan Z, Tie A, Tarnopolsky M, Bakovic M (2006). Genomic organization, promoter activity, and expression of the human choline transporter-like protein 1. *Physiological Genomics*.

[61] Yuan Z, Wagner L, Poloumienko A, Bakovic M (2004). Identification and expression of a mouse muscle-specific CTL1 gene. *Gene*.

[62] Fullerton MD, Wagner L, Yuan Z, Bakovic M (2006). Impaired trafficking of choline transporter-like protein-1 at plasma membrane and inhibition of choline transport in THP-1 monocyte-derived macrophages. *American Journal of Physiology: Cell Physiology*.

[63] Michel V, Bakovic M (2012). The ubiquitous choline transporter SLC44A1. *Central Nervous System Agents in Medicinal Chemistry*.

[64] Michel V, Singh RK, Bakovic M (2011). The impact of choline availability on muscle lipid metabolism. *Food and Function*.

[65] Schenkel LC, Bakovic M Fatty acid regulation of choline transport and glycerolipid metabolism in skeletal muscle cells.

[66] Ross BM, Moszczynska A, Blusztajn JK, Sherwin A, Lozano A, Kish SJ (1997). Phospholipid biosynthetic enzymes in human brain. *Lipids*.

[67] Houweling M, Tijburg LBM, Vaartjes WJ, van Golde LMG (1992). Phosphatidylethanolamine metabolism in rat liver after partial hepatectomy. Control of biosynthesis of phosphatidylethanolamine by the availability of ethanolamine. *Biochemical Journal*.

[68] Bleijerveld OB, Klein W, Vaandrager AB, Helms JB, Houweling M (2004). Control of the CDPethanolamine pathway in mammalian cells: effect of CTP:phosphoethanolamine cytidylyltransferase overexpression and the amount of intracellular diacylglycerol. *Biochemical Journal*.

[69] Lykidis A, Wang J, Karim MA, Jackowski S (2001). Overexpression of a mammalian ethanolamine-specific kinase accelerates the CDP-ethanolamine pathway. *Journal of Biological Chemistry*.

[70] Fullerton MD, Hakimuddin F, Bonen A, Bakovic M (2009). The development of a metabolic disease phenotype in CTP:phosphoethanolamine cytidylyltransferase-deficient mice. *Journal of Biological Chemistry*.

[71] McMaster CR, Tardi PG, Choy PC (1992). Modulation of phosphatidylethanolamine biosynthesis by exogenous ethanolamine and analogues in the hamster heart. *Molecular and Cellular Biochemistry*.

[72] Vance JE, Shiao Y-J (1996). Intracellular trafficking of phospholipids: import of phosphatidylserine into mitochondria. *Anticancer Research*.

[73] DeLong CJ, Shen Y-J, Thomas MJ, Cui Z (1999). Molecular distinction of phosphatidylcholine synthesis between the CDP- choline pathway and phosphatidylethanolamine methylation pathway. *Journal of Biological Chemistry*.

[74] Cui Z, Houweling M, Vance DE (1995). Expression of phosphatidylethanolamine N-methyltransferase-2 in McArdle-RH7777 hepatoma cells inhibits the CDP-choline pathway for phosphatidylcholine biosynthesis via decreased gene expression of CTP:phosphocholine cytidylyltransferase. *Biochemical Journal*.

[75] Walkey CJ, Donohue LR, Bronson R, Agellon LB, Vance DE (1997). Disruption of the murine gene encoding phosphatidylethanolamine N-methyltransferase. *Proceedings of the National Academy of Sciences of the United States of America*.

[76] Noga AA, Zhao Y, Vance DE (2002). An unexpected requirement for phosphatidylethanolamine N-methyltransferase in the secretion of very low density lipoproteins. *Journal of Biological Chemistry*.

[77] Vance JE, Steenbergen R (2005). Metabolism and functions of phosphatidylserine. *Progress in Lipid Research*.

[78] Kodaki T, Nikawa J-I, Hosaka K, Yamashita S (1991). Functional analysis of the regulatory region of the yeast phosphatidylserine synthase gene, PSS. *Journal of Bacteriology*.

[79] Kanfer JN, McCartney D, Hattori H (1988). Regulation of the choline, ethanolamine and serine base exchange enzyme activities of rat brain microsomes by phosphorylation and dephosphorylation. *FEBS Letters*.

[80] Nishijima M, Kuge O, Akamatsu Y (1986). Phosphatidylserine biosynthesis in cultured Chinese hamster ovary cells. I. Inhibition of de novo phosphatidylserine biosynthesis by exogenous phosphatidylserine and its efficient incorporation. *Journal of Biological Chemistry*.

[81] Kuge O, Saito K, Nishijima M (1999). Control of phosphatidylserine synthase II activity in Chinese hamster ovary cells. *Journal of Biological Chemistry*.

[82] Stone SJ, Cui Z, Vance JE (1998). Cloning and expression of mouse liver phosphatidylserine synthase-1 cDNA. Overexpression in rat hepatoma cells inhibits the CDP-ethanolamine pathway for phosphatidylethanolamine biosynthesis. *Journal of Biological Chemistry*.

[83] Stone SJ, Vance JE (1999). Cloning and expression of murine liver phosphatidylserine synthase (PSS)-2: differential regulation of phospholipid metabolism by PSS1 and PSS2. *Biochemical Journal*.

[84] Shiao Y-J, Vance JE (1995). Evidence for an ethanolamine cycle: differential recycling of the ethanolamine moiety of phosphatidylethanolamine derived from phosphatidylserine and ethanolamine. *Biochemical Journal*.

[85] Vance JE, Tasseva G (2013). Formation and function of phosphatidylserine and phosphatidylethanolamine in mammalian cells. *Biochimica et Biophysica Acta*.

[86] Jiang F, Rizavi HS, Greenberg ML (1997). Cardiolipin is not essential for the growth of *Saccharomyces cerevisiae* on fermentable or non-fermentable carbon sources. *Molecular Microbiology*.

[87] Jiang F, Ryan MT, Schlame M (2000). Absence of cardiolipin in the crd1 null mutant results in decreased mitochondrial membrane potential and reduced mitochondrial function. *Journal of Biological Chemistry*.

[88] Gohil VM, Greenberg ML (2009). Mitochondrial membrane biogenesis: phospholipids and proteins go hand in hand. *Journal of Cell Biology*.

[89] Hatch GM (2004). Cell biology of cardiac mitochondrial phospholipids. *Biochemistry and Cell Biology*.

[90] Kiyasu JY, Pieringer RA, Paulus H, Kennedy EP (1963). The biosynthesis of phosphatidylglycerol. *Journal of Biological Chemistry*.

[91] Brindley DN, Waggoner DW (1998). Mammalian lipid phosphate phosphohydrolases. *Journal of Biological Chemistry*.

[92] Choi H-S, Su W-M, Morgan JM (2011). Phosphorylation of phosphatidate phosphatase regulates its membrane association and physiological functions in *Saccharomyces cerevisiae*: identification of SER602, THR723, and SER744 as the sites phosphorylated by CDC28 (CDK1)-encoded cyclin-dependent kinase. *Journal of Biological Chemistry*.

[93] Wu W-I, Carman GM (1996). Regulation of phosphatidate phosphatase activity from the yeast *Saccharomyces cerevisiae* by phospholipids. *Biochemistry*.

[94] Kent C (1995). Eukaryotic phospholipid biosynthesis. *Annual Review of Biochemistry*.

[95] Chakraborty TR, Vancura A, Balija VS, Haldar D (1999). Phosphatidic acid synthesis in mitochondria. Topography of formation and transmembrane migration. *Journal of Biological Chemistry*.

[96] Bleijerveld OB, Brouwers JFHM, Vaandrager AB, Helms JB, Houweling M (2007). The CDP-ethanolamine pathway and phosphatidylserine decarboxylation generate different phosphatidylethanolamine molecular species. *Journal of Biological Chemistry*.

[97] Kainu V, Hermansson M, Hanninen S, Hokynar K, Somerharju P (2013). Import of phosphatidylserine to and export of phosphatidylethanolamine molecular species from mitochondria. *Biochim Biophys Acta*.

[98] Horvath SE, Wagner A, Steyrer E, Daum G (2011). Metabolic link between phosphatidylethanolamine and triacylglycerol metabolism in the yeast *Saccharomyces cerevisiae*. *Biochimica et Biophysica Acta*.

[99] Ardail D, Lerme F, Louisot P (1991). Involvement of contact sites in phosphatidylserine import into liver mitochondria. *Journal of Biological Chemistry*.

[100] Daum G, Vance JE (1997). Import of lipids into mitochondria. *Progress in Lipid Research*.

[101] Kornmann B, Currie E, Collins SR (2009). An ER-mitochondria tethering complex revealed by a synthetic biology screen. *Science*.

[102] Kornmann B, Walter P (2010). ERMES-mediated ER-mitochondria contacts: molecular hubs for the regulation of mitochondrial biology. *Journal of Cell Science*.

[103] Tan T, Ozbalci C, Brugger B, Rapaport D, Dimmer KS (2013). Mcp1 and Mcp2, two novel proteins involved in mitochondrial lipid homeostasis. *Journal of Cell Science*.

[104] Sogo LF, Yaffe MP (1994). Regulation of mitochondrial morphology and inheritance by Mdm10p, a protein of the mitochondrial outer membrane. *Journal of Cell Biology*.

[105] Lev S (2010). Non-vesicular lipid transport by lipid-transfer proteins and beyond. *Nature Reviews Molecular Cell Biology*.

[106] Vance JE (1990). Phospholipid synthesis in a membrane fraction associated with mitochondria. *Journal of Biological Chemistry*.

[107] Achleitner G, Gaigg B, Krasser A (1999). Association between the endoplasmic reticulum and mitochondria of yeast facilitates interorganelle transport of phospholipids through membrane contact. *European Journal of Biochemistry*.

[108] Zborowski J, Dygas A, Wojtczak L (1983). Phosphatidylserine decarboxylase is located on the external side of the inner mitochondrial membrane. *FEBS Letters*.

[109] Voelker DR (1991). Adriamycin disrupts phosphatidylserine import into the mitochondria of permeabilized CHO-K1 cells. *Journal of Biological Chemistry*.

[110] Jasinska R, Zborowski J, Somerharju P (1993). Intramitochondrial distribution and transport of phosphatidylserine and its decarboxylation product, phosphatidylethanolamine. Application of pyrene-labeled species. *Biochimica et Biophysica Acta*.

[111] Liu J, Dai Q, Chen J (2003). Phospholipid scramblase 3 controls mitochondrial structure, function, and apoptotic response. *Molecular Cancer Research*.

[112] Tamura Y, Onguka O, Hobbs AE, Jensen RE, Iijima M, Claypool SM (2012). Role for two conserved intermembrane space proteins, Ups1p and Ups2p, [corrected] in intra-mitochondrial phospholipid trafficking. *Journal of Biological Chemistry*.

[113] Tamura Y, Iijima M, Sesaki H (2010). Mdm35p imports Ups proteins into the mitochondrial intermembrane space by functional complex formation. *EMBO Journal*.

[114] MacDonald JIS, Sprecher H (1991). Phospholipid fatty acid remodeling in mammalian cells. *Biochimica et Biophysica Acta*.

[115] Schlame M, Ren M (2006). Barth syndrome, a human disorder of cardiolipin metabolism. *FEBS Letters*.

[116] Shindou H, Hishikawa D, Harayama T, Yuki K, Shimizu T (2009). Recent progress on acyl CoA: iysophospholipid acyltransferase research. *Journal of Lipid Research*.

[117] Malhotra A, Xu Y, Ren M, Schlame M (2009). Formation of molecular species of mitochondrial cardiolipin. 1. A novel transacylation mechanism to shuttle fatty acids between sn-1 and sn-2 positions of multiple phospholipid species. *Biochimica et Biophysica Acta*.

[118] Vreken P, Valianpour F, Nijtmans LG (2000). Defective remodeling of cardiolipin and phosphatidylglycerol in Barth syndrome. *Biochemical and Biophysical Research Communications*.

[119] Bione S, D’Adamo P, Maestrini E, Gedeon AK, Bolhuis PA, Toniolo D (1996). A novel X-linked gene, G4.5. is responsible for Barth syndrome. *Nature Genetics*.

[120] Acehan D, Vaz F, Houtkooper RH (2011). Cardiac and skeletal muscle defects in a mouse model of human Barth syndrome. *Journal of Biological Chemistry*.

[121] Zhuravleva E, Gut H, Hynx D, Marcellin D, Bleck CK, Genoud C (2012). Acyl coenzyme A thioesterase Them5/Acot15 is involved in cardiolipin remodeling and fatty liver development. *Molecular and Cellular Biology*.

[122] Yang B, Fan P, Xu A (2012). Improved functional recovery to I/R injury in hearts from lipocalin-2 deficiency mice: restoration of mitochondrial function and phospholipids remodeling. *American Journal of Translational Research*.

[123] Sparagna GC, Lesnefsky EJ (2009). Cardiolipin remodeling in the heart. *Journal of Cardiovascular Pharmacology*.

[124] Monteiro JP, Pereira CV, Silva AM (2013). Rapeseed oil-rich diet alters hepatic mitochondrial membrane lipid composition and disrupts bioenergetics. *Archives of Toxicology*.

[125] Guderley H, Kraffe E, Bureau W, Bureau DP (2008). Dietary fatty acid composition changes mitochondrial phospholipids and oxidative capacities in rainbow trout red muscle. *Journal of Comparative Physiology B*.

[126] Divakaran P, Venkataraman A (1977). Effect of dietary fats on oxidative phosphorylation and fatty acid profile of rat liver mitochondria. *Journal of Nutrition*.

[127] Yamaoka S, Urade R, Kito M (1988). Mitochondrial function in rats is affected by modification of membrane phospholipids with dietary sardine oil. *Journal of Nutrition*.

[128] Clandinin MT, Field CJ, Hargreaves K (1985). Role of diet fat in subcellular structure and function. *Canadian Journal of Physiology and Pharmacology*.

[129] Yamaoka S, Urade R, Kito M (1990). Cardiolipin molecular species in rat heart mitochondria are sensitive to essential fatty acid-deficient dietary lipids. *Journal of Nutrition*.

[130] Valianpour F, Wanders RJA, Overmars H, Vaz FM, Barth PG, van Gennip AH (2003). Linoleic acid supplemention of Barth syndrome fibroblasts restores cardiolipin levels: implications for treatment. *Journal of Lipid Research*.

[131] Aoun M, Fouret G, Michel F, Bonafos B, Ramos J, Cristol JP (2012). Dietary fatty acids modulate liver mitochondrial cardiolipin content and its fatty acid composition in rats with non alcoholic fatty liver disease. *Journal of Bioenergetics and Biomembranes *.

[132] Stavrovskaya IG, Bird SS, Marur VR (2013). Dietary macronutrients modulate the fatty acyl composition of rat liver mitochondrial cardiolipins. *Journal of Lipid Research*.

[133] Hulbert AJ, Turner N, Storlien LH, Else PL (2005). Dietary fats and membrane function: implications for metabolism and disease. *Biological Reviews of the Cambridge Philosophical Society*.

[134] Takeuchi H, Matsuo T, Tokuyama K, Shimomura Y, Suzuki M (1995). Diet-induced thermogenesis is lower in rats fed a lard diet than in those fed a high oleic acid safflower oil diet, a safflower oil diet or a linseed oil diet. *Journal of Nutrition*.

[135] Shimomura Y, Tamura T, Suzuki M (1990). Less body fat accumulation in rats fed a safflower oil diet than in rats fed a beef tallow diet. *Journal of Nutrition*.

[136] Pan DA, Storlien LH (1993). Dietary lipid profile is a determinant of tissue phospholipid fatty acid composition and rate of weight gain in rats. *Journal of Nutrition*.

[137] Pehowich DJ (1999). Thyroid hormone status and membrane n-3 fatty acid content influence mitochondrial proton leak. *Biochimica et Biophysica Acta*.

[138] Tijburg LBM, Nishimaki-Mogami T, Vance DE (1991). Evidence that the rate of phosphatidylcholine catabolism is regulated in cultured rat hepatocytes. *Biochimica et Biophysica Acta*.

[139] Morash SC, Cook HW, Spence MW (1988). Phosphatidylcholine metabolism in cultured cells: catabolism via glycerophosphocholine. *Biochimica et Biophysica Acta*.

[140] de Windt LJ, Reneman RS, van der Vusse GJ, van Bilsen M (1998). Phospholipase A2-mediated hydrolysis of cardiac phospholipids: the use of molecular and transgenic techniques. *Molecular and Cellular Biochemistry*.

[141] Zhang XH, Zhao C, Seleznev K, Song K, Manfredi JJ, Ma ZA (2006). Disruption of G1-phase phospholipid turnover by inhibition of Ca^2+^-independent phospholipase A_2_ induces a p53-dependent cell-cycle arrest in G1 phase. *Journal of Cell Science*.

[142] Mancuso DJ, Sims HF, Han X (2007). Genetic ablation of calcium-independent phospholipase A2*γ* leads to alterations in mitochondrial lipid metabolism and function resulting in a deficient mitochondrial bioenergetic phenotype. *Journal of Biological Chemistry*.

[143] Fukami K, Inanobe S, Kanemaru K, Nakamura Y (2010). Phospholipase C is a key enzyme regulating intracellular calcium and modulating the phosphoinositide balance. *Progress in Lipid Research*.

[144] Hii CST, Edwards YS, Murray AW (1991). Phorbol ester-stimulated hydrolysis of phosphatidylcholine and phosphatidylethanolamine by phospholipase D in HeLa cells. Evidence that the basal turnover of phosphoglycerides does not involve phospholipase D. *Journal of Biological Chemistry*.

[145] Minahk C, Kim K-W, Nelson R, Trigatti B, Lehner R, Vance DE (2008). Conversion of low density lipoprotein-associated phosphatidylcholine to triacylglycerol by primary hepatocytes. *Journal of Biological Chemistry*.

[146] Gao Q, Frohman MA (2012). Roles for the lipid-signaling enzyme mitoPLD in mitochondrial dynamics, piRNA biogenesis, and spermatogenesis. *BMB Reports*.

[147] Jin J-K, Kim N-H, Lee Y-J (2006). Phospholipase D1 is up-regulated in the mitochondrial fraction from the brains of Alzheimer’s disease patients. *Neuroscience Letters*.

